# An Antimicrobial Metabolite from *Bacillus* sp.: Significant Activity Against Pathogenic Bacteria Including Multidrug-Resistant Clinical Strains

**DOI:** 10.3389/fmicb.2015.01335

**Published:** 2015-12-15

**Authors:** Ajay G. Chalasani, Gunaseelan Dhanarajan, Sushma Nema, Ramkrishna Sen, Utpal Roy

**Affiliations:** ^1^Department of Biological Sciences, BITS Pilani- K. K. Birla Goa CampusGoa, India; ^2^Department of Biotechnology, Indian Institute of Technology-KharagpurKharagpur, India; ^3^Central Lab OncQuest Laboratory Pvt. Ltd. (NABL)Indore, India

**Keywords:** promising antimicrobial agent, *Bacillus* sp., multidrug resistant-bacteria, minimum inhibitory concentration, *Staphylococcus aureus*, non-haemolytic property

## Abstract

In this study, the cell free modified tryptone soya broth (pH 7.4 ± 0.2) of *Bacillus subtilis* URID 12.1 showed significant antimicrobial activity against multidrug-resistant strains of *Staphylococcus aureus, S. epidermidis, Streptococcus pyogenes* and *Enterococcus faecalis*. The partially purified antimicrobial molecule was found to be resistant to extremes of pH and temperatures and also to higher concentrations of trypsin and proteinase K. The antimicrobial molecule was purified by a three-step method that included reversed-phase high performance liquid chromatography (RP-HPLC). The minimum inhibitory concentration (MIC) values were determined for 14 species of bacteria using a microbroth dilution technique. The HPLC-purified fraction showed the MICs ranging from 0.5 to 16 μg/ml for methicillin and vancomycin-resistant *Staphylococcus aureus* (MVRSA) and methicillin-resistant *Staphylococcus epidermidis* (MRSE) strains. The molecular mass of the antimicrobial compound was determined to be 842.37 Da. The same antimicrobial fraction showed negligible haemolytic activity against human red blood cells even at a concentration as high as 100 μg/ml. Because of its significant antimicrobial activity at low MIC values coupled with its non-haemolytic property, it may prove to be a novel antimicrobial lead molecule.

## Introduction

Antimicrobial peptides (AMPs) represent a defense system against invading pathogenic bacteria and are effective therapeutically against antibiotic-resistant bacteria by causing rapid killing. Nonribosomal peptides represent a large family of bioactive secondary metabolites produced by bacteria and fungi. Many of these peptides turned out to be important antibiotics like iturin, gramicidin, and bacitracin. The production of antibiotics is considered to be a major step in enhancing the competitiveness of producing organism under an environment with limited resource (Grossman, 1995). *Bacillus* is an important genus producing an umpteen number of ribosomal and nonribosomal peptides with bioactivity (Tamehiro et al., 2002). For example, nonribosomal peptides are derived from *Bacilli* (Emmert et al., 2004; Kessler et al., 2004; Ehling-Schulz et al., 2005); peptide antibiotics are a predominant class of antimicrobial molecules produced by *B. subtilis* species (Hagelin et al., 2004; Stein, 2005). In addition, the peptides can further be modified by N-methylation, acylation, glycosylation, or heterocyclization, so they have clear advantages over ribosomally synthesized peptides in structural diversity (Von Döhren, 1995; Wu et al., 2006). *Bacilli* are especially known for their ability to produce a wide variety of bioactive compounds and wild-type *B. subtilis* strains particularly are armed with a battery of chemically diverse antimicrobial compounds which include non-ribosomally synthesized peptides (Vanittanakom et al., 1986; Von Döhren, 1995; Hagelin et al., 2004; Kessler et al., 2004; Stein, 2005; Wu et al., 2006), such as polymyxins and the lipopeptide surfactin, gene encoded bacteriocins, such as subtilosin and, within this latter group, lantibiotic, such as subtilin (Von Döhren, 1995; Stein, 2005).

The continued prevalence of methicillin-resistant *Staphylococcus aureus* (MRSA) and *S. epidermidis* (MRSE) infections represents a major clinical challenge. Interestingly last two decades have witnessed a dramatic increase of vancomycin and teicoplanin resistance of methicillin-resistant *Staphylococcus* species in several hospitals around the world (Isnansetyo and Kamei, 2003; Tabarez et al., 2006). The natural environment remains an important reservoir for microorganisms capable of producing newer potent antimicrobials (Clardy et al., 2006). A narrow-spectrum but strong antibacterial compound has been recently purified from the cell-free supernatant of soil-bacterium belonging to the genus *Bacillus* and was found to have potent antimicrobial activity against several clinically important drug-resistant pathogens including methicillin-vancomycin-resistant *Staphylococcus aureus* (MVRSA), MRSA, MRSE, and vancomycin-resistant *Enterococcus faecalis* (VRE). The present communication addresses the purification and various aspects of anti-*Staphylococcus* activity of the reversed-phase HPLC-purified fraction along with an evaluation of its overall antibacterial activities.

## Methods

### Microbial cultures, media, and growth conditions

The wild-type soil-isolate (URID 12.1) showing antimicrobial activity was grown in modified Tryptone Soya Broth (mTSB, with 0.5% yeast extract) at 37°C under shaking conditions (110 rpm). All indicator bacterial strains (Tables 1, **4**) including the quality control strains *S. aureus* ATCC 29213 and *S. aureus* MTCC 737 (ATCC 6538P) and *E. faecalis* ATCC 29212, were subcultured in Mueller-Hinton and Brain Heart Infusion (BHI) broths at 37°C. The quality control strains *S. aureus* ATCC 29213 and *E. faecalis* ATCC 29212 were obtained from OncQuest Laboratory, Indore, whereas the *S. aureus* MTCC 737 (ATCC 6538P) was procured from the Microbial Type Culture Collection (MTCC), Chandigarh, India. All the cultures used as indicator strains (Tables 1, **4**) were obtained from the MTCC, Chandigarh and National Collection of Industrial Microorganisms (NCIM), Pune, and the clinical isolates from the Central Laboratory, OncQuest Laboratory, Indore, and *S. aureus* GMC from Goa Medical College, India. The cultures were also maintained as 20% glycerol stocks at −80°C. The antimicrobial susceptibilities of the quality control strains were tested using oxacillin and vancomycin.

**Table 1:** **Antimicrobial activity of URID 12.1 by cut well agar diffusion assay using the CFS at 44 h of growth**.

**Strain number**	**Indicator organisms**	**Zone of inhibition (diameter) in mm**
1	Methicillin and vancomycin resistant *Staphylococcus aureus* 4^*^	25
2	Methicillin-resistant *S. aureus* 2^*^	22
3	*S. aureus-*GMC^*^	23
4	*S. aureus* MTCC 96	26
5	*S. aureus* MTCC 737 (ATCC 6538P)	21
6	*S. aureus* MTCC 5021	18
7	*Micrococcus luteus* MTCC 2170	25
8	Methicillin-resistant *Staphylococcus epidermidis* 3^*^	26
9	*Staphylococcus epidermidis* ATCC 12228	27
10	Vancomycin-resistant *Enterococcus faecium* 13	17
11	*Streptococcus pyogenes* MTCC 442	22
12	*S. pyogenes* MTCC 1928	16
13	*S. pyogenes* NCIM 2608	18
14	*Escherichia coli* MTCC 723	No zone
15	*Klebsiella pneumoniae*	No zone
16	*Acinetobacter baumannii* MTCC 1425	No zone
17	*Salmonella* Infantis MTCC 1167	No zone

* *Indicates clinical strains*.

### Screening for antimicrobial activity

The spot-on-lawn method was used for the bioassay of antimicrobial activity. Bacterial strain *S. aureus* MTCC 737 was used as indicator initially for the screening purpose. Soil samples were serially diluted in sterile 0.85% saline and spread to obtain single colonies. The colonies were inoculated into fresh TSB and the supernatant was collected after 24–48 h incubation. Freshly grown overnight indicator strain was mixed with soft agar (0.7%) and the mixture was transferred to plates prepoured with 1.8% agar. The supernatant was spotted, plates were incubated at 37°C for 24–48 h and inspected for the zone of inhibition. The wild-type bacterial isolate showing consistently reproducible antibacterial activity against the indicator strain was initially designated as *Bacillus* sp. URID 12 used for the construction of phylogenetic tree with the producer strain used in a separate study (Ramya et al., 2013).

### Identification of the producer strain *bacillus* sp. URID 12.1

#### PCR based identification

Genomic DNA of the isolate URID 12.1 was extracted from overnight grown culture by using the method after (Neumann et al., 1992). The genomic DNA was used to perform the 16S rRNA sequencing PCR by using the primers “fwd_name: 27F, fwd_seq: AGAGTTTGATCCTGGCTCAG, rev_name: 1492r, rev_seq: GGTTACCTTGTTACGACTT” for 16S rDNA-based identification. The amplified product was sequenced and analyzed using the NCBI BLAST. The phylogenetic tree was constructed using Mega 5.0 software (Tamura et al., 2011). Databases (GenBank) were used for sequence similarity comparison with the 16S rDNA sequence obtained. The identified strain has been redesignated as *B. sutbtilis* URID 12.1.

### MALDI-TOF (matrix-assisted laser desorption/ionization-time of flight) biotyper

#### Ethanol/formic acid extraction method

About 5–10 mg of cells was suspended in 300 μl of sterile distilled water in a micro centrifuge tube. A series of three washes were given with 900 μl absolute ethanol and 50 μl of 70% formic acid followed by centrifugation at 12000 rpm for 2 min. The pellet was dissolved in 50 μL acetonitrile and centrifuged at 12000 rpm for 2 min. One microliter from the supernatant was loaded on target and dried followed by analysis using MALDI-Biotyper Microflex, Bruker, Germany. The MALDI-biotyping for strain identification was conducted at the MTCC, Council of Scientific and Industrial Research-Institute of Microbial Technology (CSIR-IMTECH), India.

### Growth and production kinetics

The antimicrobial compound producing strain URID 12.1 was inoculated in to 100 ml of mTSB and incubated at 37°C under shaking condition. After every 4 h, 2 ml of sample was collected and centrifuged at 12000 rpm for 20 min and the supernatant was used to test the antimicrobial activity against the indicator strain *S. aureus* MTCC 96 by cut-well agar diffusion assay and the zone of inhibition in mm was noted. Simultaneously the growth was also measured spectrophotometrically at OD 600 nm and the activity was expressed as Arbitrary Unit per milliliter (AU/mL); this value is the reciprocal of the highest two-fold dilution exhibiting a zone of inhibition and estimated using the formula (2^*n*^ × 1000)/V (μl), where *n* = highest two-fold dilution showing activity and V = volume used to test antimicrobial activity.

### Antimicrobial activity spectrum

The antimicrobial compound producing strain URID 12.1 was inoculated into 100 ml of mTSB and incubated at 37°C for 44 h and the supernatant was collected by centrifugation at 12000 rpm for 20 min. The supernatant was tested for antimicrobial activity against the indicator strains (Table 1) by cut-well agar diffusion assay and the zone of inhibition in mm was noted after 24–48 h of incubation.

### Solubility in organic solvents

The supernatant (10 ml) was mixed with equal volume of organic solvents (chloroform, methanol, and n-butanol), stirred for 4 h and centrifuged at 12000 rpm for 20 min. Both the soluble and insoluble fractions were completely evaporated at 55°C, dissolved in 2 ml of sterile Milli Q water and tested for activity against the indicator strain MVRSA 4 by spot agar assay.

### Effect of pH and temperature

The effect of pH on the antimicrobial compound was determined by adjusting the supernatant pH from 1.0 to 14.0 by using 1 N HCl and 1 N NaOH and incubated at 37°C for 2 h, then neutralized to pH 8.0 before testing the activity against the indicator organisms (*S. epidermidis* ATCC 12228 and *S. aureus* MTCC 737). The effect of temperature was determined by incubating the aliquots of supernatant at 80°C for 1 h, 100°C for 30 min and autoclaving for 20 min. The untreated supernatant was used as control.

### Partial purification of the antimicrobial compound

URID 12.1 strain was grown at 37°C for 44 h in mTS broth and the cell free supernatant (CFS) was collected by centrifugation at 12000 rpm, 4°C for 20 min. The CFS was subjected to acid precipitation by adjusting the pH to 2.0 using 1 N HCl and stirring in cold room overnight (Hernández et al., 2005; Ramya et al., 2014). The precipitate was collected by centrifugation at 12000 rpm, 4°C for 20 min and dissolved in 20 mM sodium phosphate buffer pH 8.0. The antimicrobial fractions were extracted by stirring with equal volume of methanol (50% v/v) for 3 h and centrifuged to collect the supernatant (Baindara et al., 2013). The methanol was evaporated completely at 55°C and the residue was dissolved in chloroform. Adsorption chromatography was performed by using silica gel 230–400 mesh equilibrated with chloroform.

### Effect of proteolytic enzymes

Partially purified antimicrobial compound after adsorption chromatography was treated with trypsin, final concentration of 10 mg/mL at 37°C for 12 h and proteinase K, final concentration of 5 mg/mL at 55°C for 3 h (Shekh and Roy, 2012). The enzymes after incubation were inactivated by heating at 80°C for 10 min and the antimicrobial activity was tested against the indicator strain MVRSA 4. The untreated sample and the enzyme alone were used as positive and negative controls, respectively.

### Effect of surfactants

Partially purified antimicrobial compound after adsorption chromatography was incubated at 37°C for 5 h in the presence of surfactants namely Sodium dodecyl sulfate (SDS), Tween 20, Tween 80, and Triton X-100 at a final concentration of 1% (v/v) (Kayalvizhi and Gunasekaran, 2010; Ramya et al., 2014) and activity was tested against the indicator strain MVRSA 4. The untreated sample and the surfactants at a final concentration were used as positive and negative controls, respectively.

### Effect of metal salts

The partially purified antimicrobial compound obtained after adsorption chromatography was treated with metal salts (MgSO_4_, FeSO_4_, MnCl_2_, AgNO_3_, ZnSO_4_, CdCl_2_, CuSO_4_, and CaCl_2_) at a final concentration of 1 mg/mL and kept for incubation at 37°C for 1 h (Ramya et al., 2014) before testing for activity against the indicator strain MVRSA 4. Untreated sample and the metal salts at final concentration were used as positive and negative controls, respectively (Kayalvizhi and Gunasekaran, 2010).

### Sodium dodecyl sulfate (SDS)-Polyacrylamide gel electrophoresis (PAGE) and gel overlay assay

The biologically active semi purified antimicrobial compound after adsorption chromatography was subjected to 15% SDS-PAGE (Laemmli and Favre, 1973). After electrophoresis one part of the gel with molecular marker and sample was used for silver staining. The other part having sample was fixed with 25% ethanol and 5% acetic acid for 30 min. Zymogram treatments were as per Bhunia et al. (1987) and Yamamoto et al. (2003).

### Thin layer chromatography (TLC) and bioautography assay

Partially purified biologically active antimicrobial compound after adsorption chromatography was spotted on to silica gel 60 plate (7 × 4 cm; layer thickness, 0.20 mm, Merck) and developed with chloroform-methanol-water (65:25:4) as mobile phase. One part of the TLC plate was sprayed with ninhydrin (0.2%) to detect the presence of amino acids. The other part was completely dried of solvents before placing in a fresh Petri dish; the molten BHI broth was pre-inoculated separately with the indicator strains *S. aureus* MTCC 737 and MVRSA 4 along with Triphenyl tetrazolium chloride (TTC) dye, poured on each TLC plate and incubated at 37°C overnight (Tabbene et al., 2009). The R_f_ value of the antimicrobial compound was estimated. The R_f_ of the detected spots is defined as the ratio between the distance traveled by the compound divided by the distance traveled by the solvent.

### Purification of the antibacterial compound produced by *B. subtilis* URID 12.1 by HPLC fractionation

Pooled active fractions after adsorption chromatography were purified by reversed-phase high-performance liquid chromatography on a C18 column (Zorbax, 5 μm). Each run included loading a 50 μl sample to the column. HPLC-separation was performed by using acetonitrile (with 0.1% trifluoroacetic acid (TFA) and water gradient for 40 min. The gradient used was 0–50% acetonitrile for 18 min at a flow rate of 1 ml/min, 50–63% from 18 to 26 min at 0.6 ml/min, 63–68% from 26 to 38 min at 0.4 ml/min and 68–95% from 38 to 40 min at 1 ml/min. Peaks eluting from the column were detected by the diode array detection system at 210 nm. Fractions from multiple runs were pooled and tested for the antimicrobial activity against the indicator strain MVRSA 4.

### Mass spectrometry

The molecular mass of the purified compound was determined by matrix-assisted laser desorption and ionization–time of flight mass spectrometry. The mass spectrometry analysis of the HPLC-purified fraction (6A) was repeatedly performed using a Matrix-assisted Laser Desorption/Ionization-time of Flight (MALDI-TOF) mass spectrometer (UltrafleXtreme, Bruker Daltonics, Germany). An aliquot of 5 μL sample was mixed with 5 μl matrix (2,5-dihydroxy benzoic acid in acetonitrile with 0.1% TFA). The sample was spotted onto the MALDI target and air-dried. Mass spectrum was analyzed in the range of 500–3500 Da.

### Determination of minimum inhibitory concentration (MIC) and minimum bactericidal concentration (MBC)

MIC determination was performed according to the Clinical and Laboratory Standard Institute (CLSI) guidelines (CLSI, 2012). The MICs of the HPLC purified antimicrobial compound against bacterial strains were determined by broth microdilution method using BHI and cation-adjusted Mueller-Hinton broth (CA-MHB) in 96 well plates. The MICs for *S. aureus* strains were tested in CA-MH and for other strains in BHI broths since in CA-MHB the growth of *S. epidermidis* ATCC12228 and VRE strains couldn't be visualized. Resazurin dye was added to each well at a final concentration of 0.02%, as an indicator of growth. The reversed-phase HPLC purified compound was diluted by two-fold dilution in the range of 120–0.12 μg/mL and inoculated with 10^5^ CFU/mL of the indicator strains prepared using 0.5 McFarland standards. The plates with 100 μl appropriately diluted samples were incubated at 37°C with continuous shaking (110 rpm) for 25 h and the lowest concentrations at which the visible growth was not observed were recorded. The MBC was determined by streaking 10 μl of the dilutions from the incubated plates to observe the colony formation and the lowest concentration at which the colony formation was not observed were recorded.

### Haemolytic assay

Human erythrocytes were harvested from whole blood by centrifugation at room temperature for 10 min at 2000 g. The erythrocytes were washed three times with 10 mM sodium phosphate buffer pH 7.0 in 150 mM NaCl phosphate buffered saline (PBS). The pellet was resuspended in PBS to yield 20% (v/v) erythrocytes/PBS suspension. The 20% suspension was diluted 1:5 in PBS. Equal volume of the antimicrobial compound in same buffer was added at different concentrations ranging from 2.5 to 100 μg/mL and incubated for 1 h at 37°C. After incubation the dilutions were centrifuged at room temperature for 5 min at 1500 g. The OD of the supernatant was measured at 450 nm (Helmerhorst et al., 1999). Erythrocytes in PBS suspension with 1% (v/v) Tween 20 and buffer alone were used as positive and negative controls, respectively. The % haemolysis was calculated by using the formula

$$\begin{array}{l} \frac{\left(\text{A}450\;of\;peptide\;treated\;sample-\text{A}450\;of\;buffer\;treated\;sample\right)\;\times \;100}{\text{A}450\;of\;Tween\;20\;treated\;sample-\text{A}450\;of\;buffer\;treated\;sample} \end{array}$$

## Results

### Identification of the producer strain

**Figure 1:**
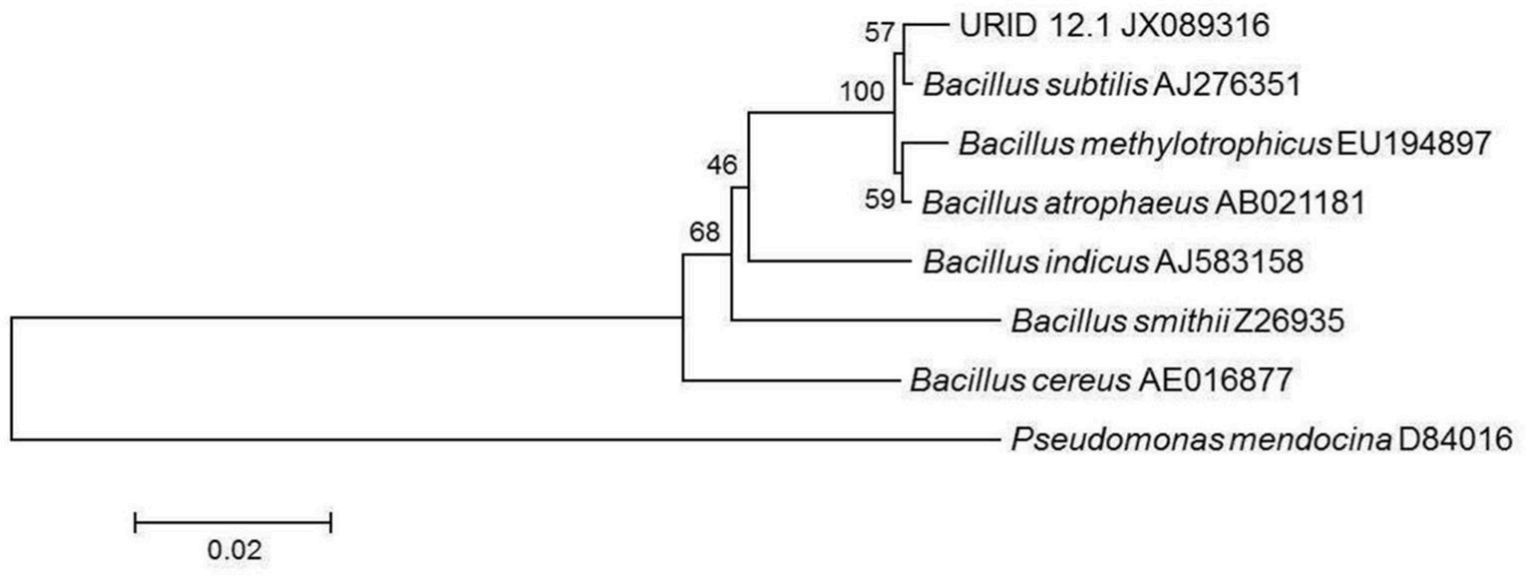
**Phylogenetic tree showing the similarity of URID 12.1 among other ***Bacillus*** species**. *Pseudomonas mendocina* was used as an outgroup.

Based on the 16S rDNA sequence (945 base pairs), the bacterial strain which is a soil-isolate responsible for the production of the bioactive metabolite was identified as *Bacillus atrophaeus* and assigned the GenBank accession number as JX156420.1 by the NCBI. The phylogenetic tree analysis using Mega 5.0 has shown the URID 12.1 more closely related to *Bacillus subtilis* (Figure 1) with bootstrapping for 1000 replicates and displaying for 100.

### Identification of the producer strain by MALDI-TOF (matrix-assisted laser desorption/ionization-time of flight) biotyper

The MALDI Biotyper identification of bacteria utilizes the ribosomal protein fingerprint of the cell and relies on high abundance proteins. The MALDI biotyping identified the producer strain as *Bacillus subtilis* with a score of 1.905 which shows the high level confidence of identification.

### Production kinetics

**Figure 2:**
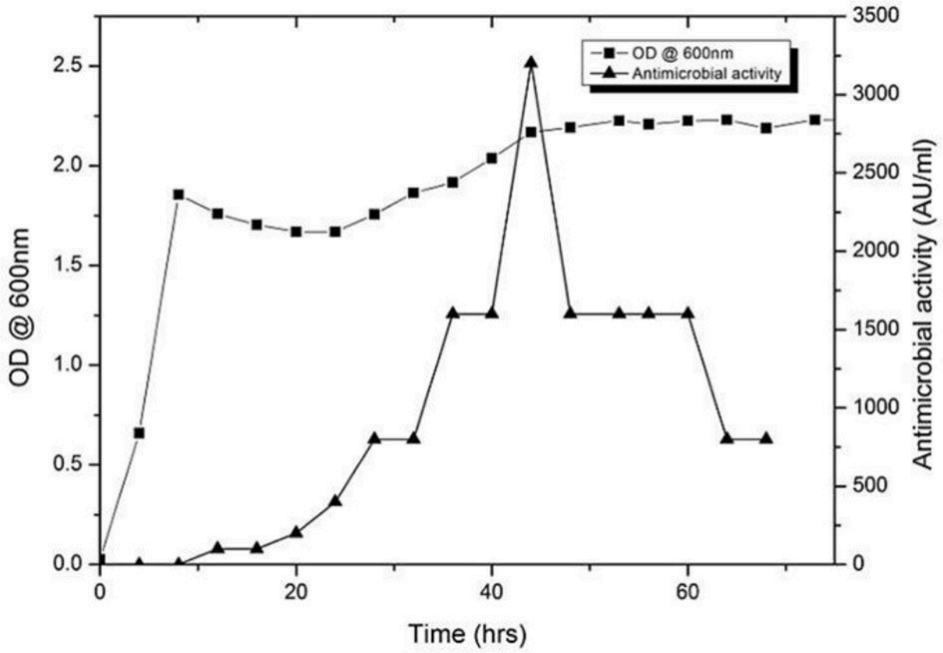
**Growth and production kinetics of URID 12.1; the antimicrobial compound production is expressed as AU/mL**.

The antimicrobial activity of the URID 12.1 was tested against the *S. aureus* MTCC 96 as the indicator strain. The production of the antimicrobial compound started at 8 h of inoculation and reached maximum by 40 h (26.0 mm); however after 48 h, the activity declined gradually (Figure 2). The maximum production of the antimicrobial compound was at the late logarithmic and early stationary phase. At 44 h the antimicrobial value was detected to be 3200 AU/mL.

### Antimicrobial activity spectrum

The sensitivity of different microbial strains to the antimicrobial compound produced by URID 12.1 was tested by cut well agar diffusion assay using the CFS at 44 h of growth (Table 1). No activity was observed against gram-negative strains (Table 1).

### Effect of pH and temperature

**Figure 3:**
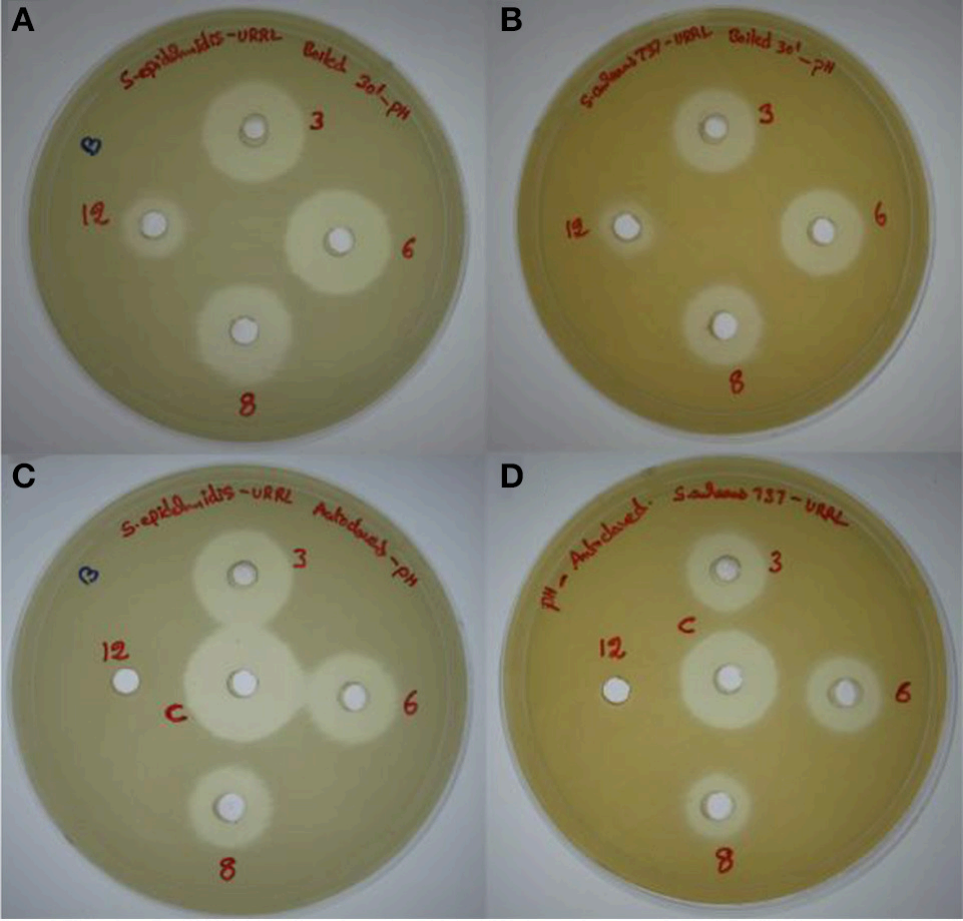
**Effect of pH (3.0, 6.0, 8.0, and 12.0) and temperatures (100 and 121°C) on the antimicrobial activity. (A,B)** Effect on antimicrobial activity after boiling for 30 min at different pH values tested against methicillin resistant *S. epidermidis* ATCC 12228 (MRSE) and *S. aureus* MTCC 737, respectively. **(C,D)** Effect on antimicrobial activity after autoclaving at different pH values against MRSE and *S. aureus* MTCC 737, respectively.

The antimicrobial compound was stable over a wide pH range from 1 to 10, and at pH 12.0 there was a slight reduction in the activity whereas there was a significant reduction in the activity at pH 14.0. The antimicrobial compound was also stable at different temperatures 80% activity was retained at 80°C for 1 h, 75% at 100°C for 30 min and 60% at 121°C for 20 min (Table 2). At acidic pH there is a slight reduction in the activity at higher temperatures (100 and 121°C) whereas at alkaline pH there was significant reduction in the activity with complete loss of activity at pH 12.0 after autoclaving (Figure 3).

**Table 2:** **Effects of pH and Temperature on the antimicrobial activity**.

**Treatment**	**Antimicrobial activity (%)**
Untreated (Control)	100
**pH**	
1.0	100
3.0	100
5.0	100
6.0	100
7.0	100
8.0	100
9.0	100
10.0	100
12.0	90
14.0	50
**TEMPERATURE**	
80°C for 1 h	80
100°C for 30 min	75
121°C for 20 min	60

### Effect of proteolytic enzymes, organic solvents, surfactants, and metal salts

The antimicrobial compound was tested for stability against proteolytic enzymes, organic solvents, surfactants, and metal salts (Table 3). The antimicrobial compound was stable after treatment with proteolytic enzymes trypsin (10 mg/mL) and proteinase K (5 mg/mL). The antibacterial activity against MVRSA 4 was retained by trypsin and proteinase K treated samples. The antimicrobial activity was not reduced by organic solvents (50% v/v) and the surfactants at a final concentration of 1%, indicating the hydrophobic nature of the compound. The biological activity was not affected by the presence of metal salts (Table 3).

**Table 3:** **Effect of enzymes, solvents, surfactants, and metal salts**.

**Treatment**	**Concentration**	**Activity**
Trypsin	10 mg/mL	+
Proteinase K	5 mg/mL	+
Surfactants (Tween 20, Tween 80, and Triton X-100)	1% (v/v)	+
Organic solvents (chloroform, methanol, n-butanol)	50% (v/v)	+
Metal salts (MgSO_4_, FeSO_4_, MnCl_2_, AgNO_3_, ZnSO_4_, CdCl_2_, CuSO_4_, and CaCl_2_)	1 mg/mL	+

*+, indicates the retention of activity*.

### PAGE and gel overlay assay

**Figure 4:**
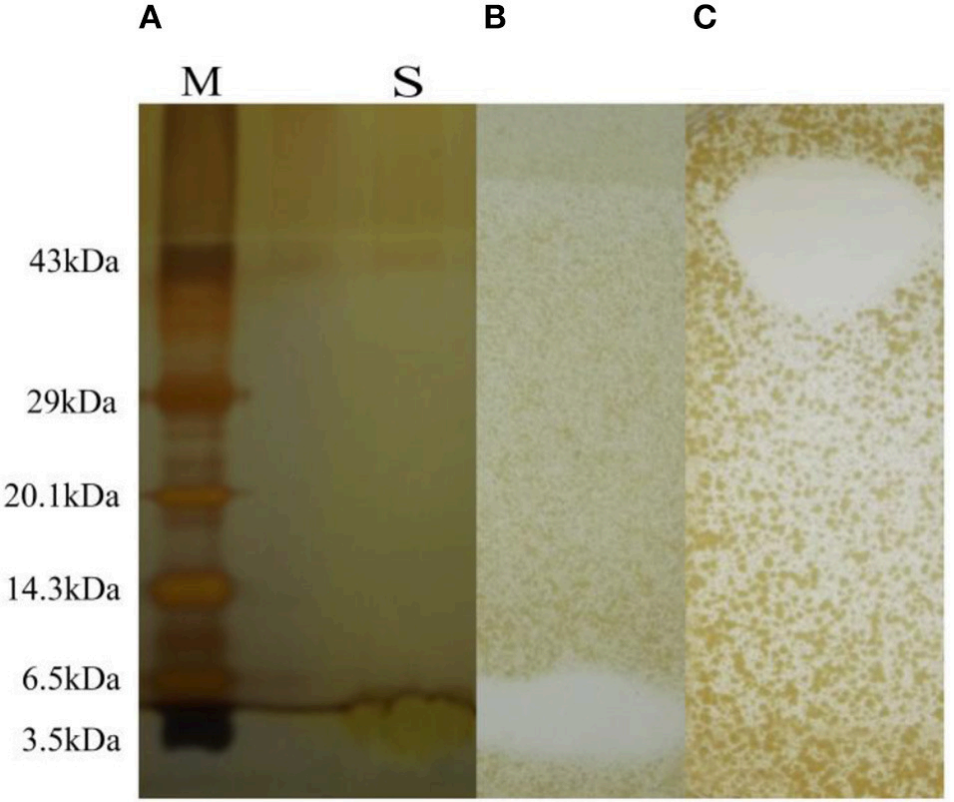
**(A)** SDS-PAGE with molecular marker (lane M) and sample (lane S). **(B)** Antimicrobial activity shown as zone of inhibition after SDS-PAGE and zymogram. **(C)** Antimicrobial activity shown by Native PAGE and zymogram.

In SDS-PAGE the band less than 3.0 kDa has shown antimicrobial activity (Figures 4A,B) against *S. aureus* MTCC 737. In the Native PAGE the activity was observed at the top of the resolving gel indicating may be the net positive charge of the antimicrobial compound or aggregation in its native form (Figure 4C). Zymogram showed activity against *S. aureus* MTCC 737.

### Thin layer chromatography (TLC) and bioautography assay

**Figure 5:**
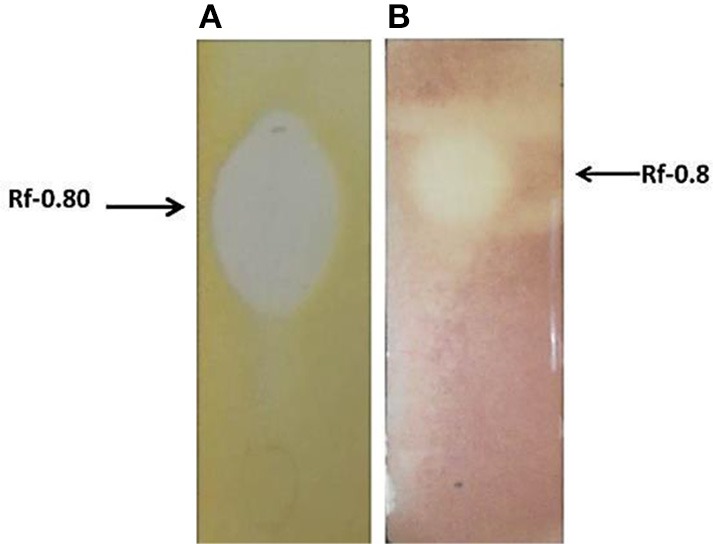
**(A)** Bioautography assay showing zone of inhibition, against *S. aureus* MTCC 737. **(B)** Bioautography assay (using the TTC dye) showing antimicrobial activity against MVRSA 4 showing an Rf value 0.80.

One part of the TLC plate was used for bioautography assay and showed clear zone of inhibition (Figures 5A,B). The region corresponding to the antimicrobial compound when sprayed with 0.2% ninhydrin showed negative result. This may be due to blocking of the N-terminal end by modification. The Rf value of the spot was 0.8 (Figures 5A,B), the center of zone of inhibition was taken as the distance traveled by the solute.

### Purification of the antimicrobial compound

**Figure 6:**
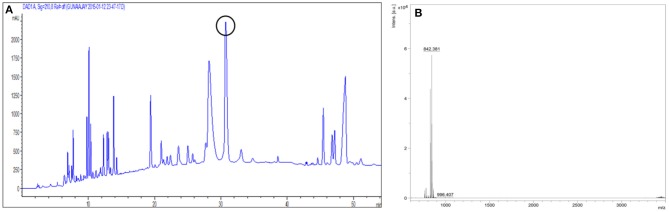
**(A)** Reversed Phase HPLC chromatogram wherein the antimicrobial compound had a retention time of 30.5 min (encircled peak). **(B)** MALDI-TOF analysis showing the molecular mass as 842.37 Da.

After acid precipitation the antimicrobial fractions were extracted with methanol and adsorption chromatography was performed using silica gel 230–400 mesh. Active fractions were further purified by RP-HPLC on a C18 column; the eluting active fraction showed a retention time of 30.5 min (Figure 6A, encircled peak), indicating its non-polar nature.

### Mass spectrometry

The molecular mass of the purified compound determined by MALDI was 842.37 Da (Figure 6B).

### Determination of minimum inhibitory concentration (MIC) and minimum bactericidal concentration (MBC)

MIC of the HPLC purified antimicrobial compound against different strains was calculated using two-fold micro broth dilution method in 96 micro-well plates. The MIC values against different strains were in the range of 0.3–16 μg/mL (for MRSA 5) in BHI and CA-MHB to 120 μg/mL (for *S. pyogenes* NCIM 2608). The MIC and MBC values determined against *S. epidermidis* ATCC 12228 was 2 and 4 μg/mL only in BHI broth. The MBC values were in the range of 2–120 μg/mL (Table 4). The MIC values determined against *E. faecalis* ATCC 29212 and multidrug-resistant VRE were 64 and >64 μg/mL, respectively.

**Table 4:** **MIC and MBC values against different indicator strains in comparison with Subtilosin A, Sublancin 168, and S70-2**.

**Indicator strain**	**MIC in BHI**	**MBC**	**MIC in CA-MHB**	**MBC**	**Sublancin 168**	**Subtilosin A**	**S70-2**
*S. aureus* ATCC 29213	–	–	8.0	16.0	–	–	250.0
*S. aureus* MTCC 737 (*S. aureus* ATCC 6538P)	0.3	7.5	4.0	8.0	–	100.0	–
Methicillin resistant *S. aureus* 2 [Barcode ID 1401101153]^*^	15.0	30.0	8.0	64.0	–	–	–
Methicillin and vancomycin resistant 4 *S. aureus* [Barcode ID. 1312105888]*/S. aureus* 12600^€^	0.5	2.0	8.0	16.0	>100^€^	–	–
Methicillin resistant *S. aureus* 5 [Barcode ID 1312105847]^*^	–	–	16.0	64.0	–	–	–
*S. aureus* GMC^*^	1.0	3.75	–		–	–	–
*S. epidermidis* ATCC 12228	2.0	4.0	–	–	No activity	–	–
Methicillin resistant *S. epidermidis* 3 [1401101084]^*^	1.0	15.0	2.0	16.0	–	–	–
*S. pyogenes* NCIM 2608/*S. pyogenes* ATCC 19615^€^	120.0	120.0	–		–	1.25	–
*S. pyogenes* MTCC 1928/*S. pyogenes* 49399^€^	60.0	>60		–	100.0^€^	–	–
*M. luteus* MTCC 2170	0.3	15.0	–	–	–	–	–
*Bacillus* wild-type culture^**^	30.0	120.0	–	–	–	–	–
*E. faecalis* ATCC 29212	64.0	–	–	64.0	–	–	–
Vancomycin and methicillin resistant *E. faecium* 13 [Barcode ID 1312103662]^*^/*E. faecalis* ATCC 29219^€^/*E. faecalis* 19433^▴^	>64.0	128.0	–	>64	No activity^▴^	–	62.5

*All values are expressed in (μg/mL)*.

* *Clinical isolates were identified by an automated analyser Phoenix-100 (Becton Dickinson)*.

–*Denotes “Not Determined.”*

€, ▴ *Denotes the MIC produced by the antimicrobial substance against the indicator strain used shown in the first column*.

** *Environment isolate*.

### Haemolytic assay

**Figure 7:**
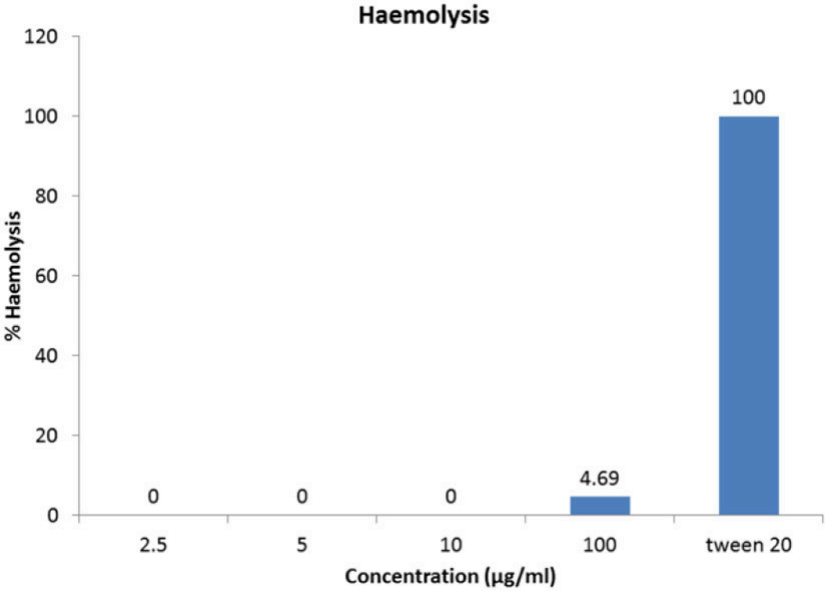
**Haemolytic activity of antimicrobial compound**. One-hundred microgram per milliliter of the HPLC-purified antimicrobial fraction showed merely 4.69% hemolysis to cause any adverse haemolytic effects.

As the antimicrobial compounds are used for biomedical applications in treating infectious diseases it is important to determine the extent of haemolysis on freshly drawn human red blood cells. The antimicrobial compound has shown 4.69% haemolysis at 100 μg/ml concentration (Figure 7). Haemolysis obtained by 1% Tween 20 was considered 100% haemolysis (Figure 7).

## Discussion

The current observations from the literature survey indicate that antibiotic resistance is rapidly evolving toward most known antibiotics and the development of alternative antibiotics is lagging far behind to combat serious infectious diseases.

The semi-purified antimicrobial substance was subjected to various types of physico-chemical tests. After heat, acid, and alkaline treatment, the inhibition zones produced by the partially purified antimicrobial compound remained almost the same (Table 1). This observation indicated that antimicrobial compound was very stable. Similar observations were reported in several cases, for example, Bacillocin Bb produced by the *Brevibacillus brevis* Bb from soil (Faheem et al., 2007; Saleem et al., 2009) is a bacteriocin like inhibitory substance (BLIS) that is stable within the pH range of 1.0–9.0, resistant to heat (100°C for 30 min), as well as detergents and organic solvents. The adsorption chromatography- purified antimicrobial substance in the present study also demonstrated resistance to surfactants and organic solvents (Table 3).

The antimicrobial spectrum, pH-tolerance, thermal stability, and resistance to proteolytic enzymes displayed by the antimicrobial compound produced by URID 12.1 resemble those of several other *Bacillus*-derived antibacterial peptides, such as bacillocin Bb (Faheem et al., 2007; Saleem et al., 2009), polyfermenticin SCD (Lee et al., 2001), pumilicin (Aunpad and Na-Bangchang, 2007), brevicin AF01 (Guo et al., 2012). It was reported recently (Guo et al., 2012) that the crude extract of *Paenibacillus* OSY-SE was resistant to heat and changes in pH; most of its antimicrobial activity was retained after holding at 80°C for 24 h, autoclaving at 121°C for 5 min, and exposure to pH 3.0, 5.0, and 9.0. Paenibacterin was resistant to treatment with trypsin but not the other proteolytic enzymes like pronase (Guo et al., 2012).

MRSA strains often causing nosocomial infections develop resistance to antimicrobial compounds by modifying its cell surface teichoic acid with D-alanine and show increased MIC (Peschel and Collins, 2001; Peschel, 2002). In the present study, altogether 11 Gram-positive bacteria tested were sensitive to HPLC-purified antibacterial compound. The values of the MICs were presented in Table 4. It is worth observing that the MICs were comparable to the MICs produced by many antimicrobial substances produced by *Bacillus*.

Out of 14 strains tested for the determination of MIC values, the methicillin and vancomycin resistant *S. aureus* (MVRSA) strain 4 was β-lactamase positive and resistant to amoxicillin/clavulanate, cefoxitin (>8 μg/ml), ciprofloxacin, erythromycin, mupirocin, and teicoplanin, and vancomycin (>16 μg/ml); another clinical isolate MRSA 5 was β-lactamase positive and cefoxitin (>8 μg/ml) resistant. The MIC value determined was 0.5 μg/ml whereas the MBC value was 2.0 μg/ml for MVRSA 4. High prevalence of mupirocin resistance in *S. aureus* isolates from a pediatric population as a result of mupirocin exposure in some areas of New York was reported underlining the importance of antimicrobial substance effective against mupirocin-resistant *S. aureus* (Antonov et al., 2015). Likewise, the MRSA strain 2, resistant to penicillin, amoxicillin/clavulanate, cefoxitin (>8 μg/ml), gentamicin (high level 500 μg/ml), clindamycin, ciprofloxacin, and erythromycin, the MIC values determined against it were 15.0 and 8.0 μg/ml in BHI and CA-MHB, respectively. The purified antimicrobial substance showed its strong antimicrobial potential as reflected by low MIC values of 1.0 and 2.0 μg/ml using BHI and CA-MHB against the MRSE 3 (Table 4) which besides being β-lactamase-positive is also resistant to cefoxitin and penicillin, and against the *S. epidermidis* ATCC 12228, the MIC value was determined as 2.0 μg/ml in BHI broth (Table 4). The data analysis of the MIC values (Table 4) clearly indicate either superior or comparable results for the purified antimicrobial compound from the URID 12.1 when compared to *Bacillus* derived antimicrobial compounds namely Subtilosin A, Sublancin 168, and S70-2 (Paik et al., 1998; Shelburne et al., 2007; Tabbene et al., 2010). The antimicrobial compound from URID 12.1 showed similar MIC and MBC values for two *S. pyogenes* strains. For four *S. aureus* strains used in the study, the MIC and MBC ratios are no more than 1:4 (Table 4) indicating the compound's potential to act as an antibacterial agent since any antibacterial agent with an MBC no more than four times the MIC is usually regarded as bactericidal (French, 2006).

Our result also showed that the HPLC-purified antimicrobial compound had negligible hemolytic activity against 2% human blood red cells even at 100 μg/ml concentration (Table 4). In this context, it is worth mentioning that out of 14 strains used in the present study, for 11 strains, the MIC values recorded were less than or equal to 30 μg/ml. The lichenicidin from *B. licheniformis* DSM 13 was reported to have inhibited *S. aureus, S. pyogenes, B. subtilis* but neither caused hemolysis nor inhibited the growth of gram-negative bacteria (Dischinger et al., 2009). Lichenicidin might not be a lipopeptide antibiotic that generally causes hemolysis (Volpon et al., 1999; Leclère et al., 2005). The lack of haemolytic activity might indicate that the antimicrobial compound is probably devoid of haemolytic/cytotoxic activity; the same inference was drawn for the S07-2 which lacked haemolytic activity even at the concentration of 1000 μg/ml (Tabbene et al., 2010). Our study has unequivocally established the fact that the antimicrobial compound although possesses a narrow-spectrum of antibacterial activity yet it has high antibiotic potential; however further studies are needed to determine the *in vivo* efficacies and cytotoxic effects for their utility in clinical applications. After a critical reconnoitering of molecular masses and the antimicrobial profiles and potentials of all the previously reported *Bacillus*-derived antimicrobial compounds, no anti-*Staphylococcus* and anti-*Streptococcus pyogenes* compound of molecular mass of range between 800 and 850 Da with a low MIC values and high proteolytic stability was found to be reported and thus promises to be a novel antimicrobial principle. To the best of our knowledge, an antimicrobial agent or principle possessing significant activity against multidrug-resistant strains and molecular weight as low as 0.84 kDa has not been reported earlier from any wild-type *Bacillus* sp.

## Funding

Authors express their gratitude to the director of BITS Pilani-K.K. Birla Goa Campus and the Vice Chancellor for providing BITS-University Annual contingency grant which has been partly utilized for this investigation.

### Conflict of interest statement

The authors declare that the research was conducted in the absence of any commercial or financial relationships that could be construed as a potential conflict of interest.
